# A monogenic dominant mutation in *Rom1* generated by N-ethyl-N-nitrosourea mutagenesis causes retinal degeneration in mice

**Published:** 2010-03-10

**Authors:** Hajime Sato, Tomohiro Suzuki, Kyoko Ikeda, Hiroshi Masuya, Hideki Sezutsu, Hideki Kaneda, Kimio Kobayashi, Ikuo Miura, Yasuyuki Kurihara, Shunji Yokokura, Kohji Nishida, Makoto Tamai, Yoichi Gondo, Tetsuo Noda, Shigeharu Wakana

**Affiliations:** 1 Department of Ophthalmology, Tohoku University Graduate School of Medicine, Sendai, Japan; 2 Technology and Development Team for Mouse Phenotype Analysis, RIKEN BioResource Center, Ibaraki, Japan; 3 Technology and Development Unit for Knowledge Base of Mouse Phenotype, RIKEN BioResource Center, Ibaraki, Japan; 4 Transgenic Silkworm Research Center, National Institute of Agrobiological Sciences, Ibaraki, Japan; 5 Department of Environment and National Science, Graduate School of Environment and Information Science, Yokohama National University, Yokohama, Japan; 6 Mutagenesis and Genomics Team, RIKEN BioResource Center, Ibaraki, Japan; 7 Team for Advanced Development and Evaluation of Human Disease Models, RIKEN BioResource Center, Ibaraki, Japan

## Abstract

**Purpose:**

To characterize an N-ethyl-N-nitrosourea-induced dominant mouse mutant, *M-1156*, that exhibits progressive retinal degeneration and to investigate the pathogenesis of the retinal phenotype in the mutant.

**Methods:**

A positional candidate gene approach was used to identify the causative gene in the *M-1156* mutant. Funduscopic examination, light microscopy, transmission electron microscopy, and electroretinography were performed to analyze the *M-1156* phenotype. Real-time quantitative PCR, immunohistochemistry, and western blotting were also performed.

**Results:**

Linkage analysis enabled the mutant gene to be mapped to a region of chromosome 19 containing *Rom1*, which encodes rod outer segment membrane protein 1. Sequence analysis demonstrated that the mutation consisted of a single base T→C substitution at position 1,195 in *Rom1* (M96760, National Center for Biotechnology Information [NCBI]) and that the mutant allele was expressed. A putative missense mutation designated *Rom1^Rgsc1156^* that was identified in the *M-1156* mutant mouse causes a Trp to Arg substitution at position 182 in the translated protein. *Rom1^Rgsc1156^* heterozygotes were found to have a mottled retina and narrowed arteries in the fundus oculi. Photomicrographs of the retina revealed significant differences among the genotypes in the thickness of the outer nuclear layer and in the length of the outer segments of the photoreceptors. The alterations were more marked in the homozygotes than in the heterozygotes. Electron micrographs showed that the diameters of the discs varied in the heterozygotes and that the discs were more compactly stacked than in the wild type. There were significant differences among the genotypes in the amplitude of the a-wave in single-flash electroretinograms, but there were no significant differences among the photopic electroretinograms. Real-time quantitative PCR showed that there were no significant differences among the genotypes in *Rom1* or peripherin/rds (*Prph2*) mRNA levels relative to the rhodopsin (*Rho*) mRNA level. Rom1 and Prph2 immunoreactivity were decreased in the retinas of the *Rom1^Rgsc1156^* mutants. Semiquantitative western blot analysis of retinas from 3-week-old *Rom1^Rgsc1156^* mutants demonstrated significant decreases in Rom1, Prph2, and Rho protein levels in all of the genotypes.

**Conclusions:**

The Trp182Arg substitution in *Rom1^Rgsc1156^* mutants causes retinal degeneration. The results suggested that Trp182Arg mutant Rom1 causes a decrease in the levels of wild-type Prph2 and Rom1, which in turn cause a reduction in the level of Prph2 containing tetramers in the disc rim region and ultimately result in unstable, disorganized outer segments and photoreceptor degeneration.

## Introduction

Retinitis pigmentosa (RP) is a genetically heterogeneous disorder that may be inherited as an autosomal dominant, autosomal recessive, or X-linked trait [1] and it affects about one in 5,000 individuals worldwide. RP patients progressively lose vision because both rods and cones throughout the retina gradually die. Mutations of genes that are mainly expressed in photoreceptors and retinal pigment epithelium are associated with RP, and phenotypic severity depends on the causative gene.

Rod outer segment membrane protein 1 (Rom1) and peripherin/rds (Prph2), which are structurally similar, are localized along the rim region of photoreceptor discs. They are 35% identical at the amino acid level and contain four putative membrane-spanning domains, a large intradiscal loop that connects the third and fourth transmembrane domains, and a long C-terminal segment [2-4]. *rds* mice that are homozygous for a null mutation in peripherin/rds (*Prph2*) fail to develop outer segments, and highly disorganized structures form in heterozygous *rds* mice; homozygous *Rom1* knockout mice develop outer segments with only mild defects [5-10]. Thus, Prph2 is likely to play a more important role than Rom1 in the formation and stabilization of photoreceptor outer segments.

Mutations in peripherin/retinal degeneration, slow (*PRPH2*) cause hereditary human retinal degenerative diseases that are characterized by autosomal dominant RP and macular dystrophy [11-14], and most of the pathological changes are localized within the large intradiscal loop. By contrast, there has been no clear evidence thus far that mutations in retinal outer segment membrane protein 1 (*ROM1)* alone cause retinal degeneration [15], although it is well known that only persons who are double heterozygous for *ROM1* and *PRPH2* develop RP in a digenic inheritance pattern [16].

N-ethyl-N-nitrosourea (ENU) is a powerful chemical mutagen that induces random point mutations in genomic DNA with high frequency [17-20]. Phenotype-based screening enables comprehensive collection of mutations in genes involved in specific biologic pathways. Because human genetic diseases are often caused by point mutations rather than gross alterations of the genome, ENU mutagenesis is suitable for production of mutants that can serve as models of human disease [21-24].

Our ENU mutagenesis project has yielded several dominantly inherited mouse mutations that are manifested by retinal and/or optic disc abnormalities, and one of the mutant mice manifested progressive retinal degeneration. A positional candidate gene approach enabled a point mutation in *Rom1* to be identified as the cause of this phenotype. The results of this study provide evidence that a monogenic dominant mutation in *Rom1* induces retinal degeneration. We analyzed the phenotype of the mutants in detail, and in this article we discuss potential implications for the pathogenesis of retinal degeneration.

## Methods

### Generation and phenotype-based screening of mice for N-ethyl-N-nitrosourea-induced mutants

We performed the animal studies under the guidelines issued by the RIKEN BioResource Center (Tsukuba, Japan) in the “Outline for conducting animal experiments” (issued August 1999, revised October 2001). We obtained stock mice from CLEA Japan, Inc. (Tokyo, Japan). The method used for mouse ENU mutagenesis is available at RIKEN BioResource Center ENU Mutants website and has been described previously [20-24]. At 8–10 weeks of age, C57BL/6J male mice were intraperitoneally injected with 85 mg/kg or 100 mg/kg bodyweight ENU (Sigma, Tokyo, Japan) twice at weekly intervals. The injected male mice were mated with DBA/2J female mice after a sterile period (approximately 10–11 weeks). Phenotypic screenings were routinely performed on the first generation (G1) progeny. Funduscopy was performed at 45 weeks of age. Phenodeviant individuals (mutant candidates) were assigned M numbers.

### Funduscopic examination

Pupils were dilated with 0.5% tropicamide and 0.5% phenylephrine (Santen Pharmaceutical Co., Ltd., Osaka, Japan) diluted 5–7.5 fold with physiologic saline. The fundi were examined by indirect ophthalmoscopy, and photographs were taken by using a GENESIS hand funduscope camera (Kowa Co., Ltd., Nagoya, Japan) with a Volk 90D condensing lens (Volk Optical, Mentor, OH), as described [25-27]. In short, the camera was fitted to a dissection microscope base for stability and a foot pedal was used to operate the shutter. Photographs were taken using conscious mice. One hand was used to hold the mouse while two fingers of the other hand were used to retract the eyelids. We used the condensing lens mounted between the camera and eye. The mouse was held on its side on the microscope platform beneath the lens. Focusing was achieved by moving the mouse.

### Inheritance testing and gene mapping

To test for phenotype transmission and to genetically map the causative gene, we backcrossed M-1156 with DBA/2J animals and recovered 125 backcross (N2) progeny. Because it was difficult to determine whether 16 of the 125 mice had abnormal fundi at 25 weeks of age, they were not used in the chromosome mapping analysis. Genomic DNA was prepared from the tail tips of the 109 remaining backcross progeny by using the NA-2000 automatic nucleic acid isolation system (KURABO, Osaka, Japan). We used the db-SNP website for single nucleotide polymorphisms (SNPs) and microsatellite markers listed on the Mouse Genome Informatics (MGI website) for simple sequence length polymorphisms. After examining 118 polymorphic markers spaced evenly throughout the genome by the TaqMan MGB assay (Applied Biosystems, Tokyo, Japan) and ABI 7700 and ABI 7900 sequence detection systems, five markers (D19Mit109, D19Mit42, D19Mit69, D19Mit110, and rs3023477) on chromosome 19 were chosen for fine mapping of the causative gene.

### Search of the *M-1156* genome for the causative mutation

We used Positional MEDLINE (PosMed), a semantic Web system, to provide a list of ranked candidate genes within the region [28,29] to search for the causative mutation in the mapped chromosomal region. We designed primer pairs based on the genomic sequence of *Rom1* (NC_000085) and sequenced the region from –110 bases upstream to 1,889 bases downstream of the *Rom1* gene to search for mutations of the *M-1156* genome.

### Genotyping of the *M-1156 Rom1* allele

The *Rom1^Rgsc1156^* allele was genotyped by allele-specific primer-PCR methods. The wild-type-specific primer (5′-AAG TAA CGG TTG CTG ACC GA-3′) or the Rgsc1156-specific primer (5′-AAG TAA CGG TTG CTG ACC GG-3′) was paired with a common opposite-strand primer (5′-GCT TTG CCA GTA AGT CTC AA-3′).

### Electroretinogram

Single-flash electroretinograms (ERGs) of both eyes of *Rom1^Rgsc1156^* mutant mice were obtained at 7, 35, and 50 weeks of age. Mice were dark adapted overnight and then prepared for recording under dim red light. The mice were anesthetized with an intraperitoneal injection of physiologic saline containing ketamine (80 mg/kg; Daiichi Sankyo Propharma Co., Ltd., Tokyo, Japan) and xylazine (16 mg/kg; Bayer Medical Ltd., Tokyo, Japan). Their pupils were dilated with 0.5% tropicamide and 0.5% phenylephrine, and their corneas were anesthetized with 0.4% oxybuprocaine hydrochloride (Santen Pharmaceutical Co., Ltd.) to prevent blinking. Mice were placed prone on a temperature-regulated heating pad throughout the recording session. After coating the cornea with drops of 1.5% hydroxyethylcellulose gel (Senju Pharmaceutical Co., Ltd., Osaka, Japan), a 2.8-mm diameter customized white-light-emitting diode (LED) electrode (Mayo Corporation, Ltd., Inazawa, Japan) was placed on the surface of the cornea with a micromanipulator and used as a recording electrode. A stainless-steel needle was attached subcutaneously to the nasal bone as a reference electrode, and another stainless-steel needle was inserted subcutaneously near the tail as a ground electrode. The flash stimulus was controlled by a white LED luminescent device (LS-W: Mayo Corporation, Ltd.). Electrical signals were amplified with a Dual Bio Amp (Model ML135; ADInstruments Pty. Ltd., New South Wales, Australia) at a gain of 4,000 and bandpass filtered between 0.3 and 500 Hz (–3 dB points). Signals were digitized at a rate of 10 kHz with a data acquisition device (PowerLab2/25, Model ML825; ADInstruments Pty. Ltd.). Stimulus presentation and data acquisition were programmed and performed with data recording and analysis software (Scope v3.7; ADInstruments Pty. Ltd.). Flash stimuli were 5 ms in duration and had an intensity of 1,000 cd/m^2^ measured at the eye. A total of five responses obtained at 1–min intervals were averaged for each eye. The amplitude of the a-wave was measured from the prestimulus baseline to the a-wave trough. The amplitude of the b-wave was measured from the a-wave trough to the peak of the b-wave or, if the a-wave was not measurable, from the prestimulus baseline.

Photopic ERGs were obtained from single eyes of *Rom1^Rgsc1156^* mutant mice at 7 weeks of age. Light-adapted responses were recorded with white flashes (5 ms duration and 1,000 cd/m^2^ intensity) on the rod-saturating background (25.1 cd/ m2) after 10 min of exposure to the background light to allow complete light adaptation. A total of 16 responses obtained at 1-s intervals were computer averaged for each eye.

### Light microscopy

Anesthetized mice with an intraperitoneal injection of physiologic saline containing pentobarbital sodium (90 mg/kg; Kyoritsu Seiyaku Co., Ltd., Tokyo, Japan) were intracardially perfused with 4% paraformaldehyde (Nacalai Tesque, Inc., Kyoto, Japan) /2.5% glutaraldehyde (Wako Pure Chemical Industries, Ltd., Osaka, Japan) in 0.1 M phosphate buffer (PB). Eyes were enucleated and fixed in fresh fixing buffer for 1 day at room temperature and then dehydrated by immersion in a series of increasing ethanol concentrations. After three changes of xylene, the eyes were embedded in paraffin wax. A mid-horizontal series of sections (3-µm thick) through the optic nerve head of each eye was prepared. The sections were stained with hematoxylin and eosin (Muto Pure Chemicals Co., Ltd., Tokyo, Japan) by the standard protocol and viewed with an AX80 microscope (Olympus Inc. Tokyo, Japan). The thickness of the outer nuclear layer and inner nuclear layer and the length of the outer segment of the photoreceptors were measured by light microscopy at the point where the ratio of the distance from the optic disc to the point to the distance from the optic disc to peripheral end of the retina was 0.2, as described elsewhere [30].

### Transmission electron microscopy

Anesthetized mice were intracardially perfused with 2% paraformaldehyde/2.5% glutaraldehyde in 0.1 M PB. Eyes were enucleated and fixed in fresh fixing buffer for 1 day. The anterior segments of the eyes were removed, and the eyecups were postfixed for another day. After rinsing the eyecups, eyecups were immersed for 1 day in 0.1 M PB at 4 °C. The eyecups were osmicated for 2 h in 1% aqueous solution of osmium tetroxide (TAAB Laboratories Equipment Ltd., Aldermaston, Berks, England) at 4 °C and then dehydrated through ascending ethanol concentrations (50%, 70%, 80%, 90%, and 100% for 30 min each). After passing the specimens through propylene oxide (Wako Pure Chemical Industries, Ltd., Osaka, Japan) twice for 1 h each time, the specimens were embedded in pure Epon (TAAB Laboratories Equipment Ltd.) for 2 days at 60 °C. Ultrathin sections were cut with a microtome (U-5; LBK Instruments Ltd., Bromma, Sweden), and the sections (80–100 nm) were stained with saturated uranyl acetate and Reynolds lead citrate (Wako Pure Chemical Industries, Ltd.). The sections were examined with an electron microscope at 80 kV (JEM-100S; JEOL, Tokyo, Japan).

### Preparation of anti-Rom1 and anti-Prph2 monoclonal antibodies

A synthetic 13-mer for Rom1 (LAHYKDTEVPGRC) and a 20-mer for Prph2 (CKSNQVEAEGADAGPAPEAG), corresponding to amino acids 141–153 in mouse Rom1 and 328–346 in mouse Prph2, respectively, were purchased from Invitrogen (Carlsbad, CA). The peptides were conjugated with keyhole limpet hemocyanin (KLH; Pierce, Rockford, IL) and injected into BALB/ cAJcl mice four times at 2-week intervals. Three days after the final booster shot, lymph node cells were fused with myeloma line P3U1 cells. The cells were cultured in GIT medium (Nihon Pharmaceutical Co. Ltd., Tokyo, Japan) for 14 days, and the culture supernatants were screened first by Enzyme-Linked ImmunoSorbent Assay (ELISA) against immunized peptides. Then, the positive samples were subjected to western blot analysis for extracts from Cos-7 cells overexpressing either Rom1 or Prph2. Hybridoma cells from the positive wells were cloned by the standard limiting dilution technique [31]. Anti-Rom1 (clones 1–38) and anti-Prph2 (clones 7–27) were used in this study.

### Immunohistochemistry

Anesthetized mice were intracardially perfused with 4% paraformaldehyde in ice-cold 0.1 M PB, and the eyes were enucleated and fixed for 1 day at 4 °C. The anterior segments were removed, and the eyecups were immersed in a graded series of sucrose solutions (10% W/V, 20%, and 30%) in PBS (8.10 mM NaHPO_4_, 1.47 mM KHPO_4_, 137 mM sodium chloride, 2.68 mM KCl, pH 7.4) at 4 °C for 1 day per step; the eyecups were then embedded in Tissue-Tek OCT Compound (Sakura Finetek, Tokyo, Japan) and frozen in acetone at dry-ice temperature. A mid-horizontal series of 10-µm-thick sections through the optic nerve head of each eye was prepared, and the sections were allowed to dry in air. After incubation in 0.3% hydrogen peroxide (H_2_O_2_) for 10 min and rinsing in PBS for 5 min, the sections were blocked for 30 min with 1% bovine serum albumin (BSA) in PBS and washed three times with PBS for 5 min each time. The sections were then incubated for 16 h at 4 °C with Rom1 or Prph2 monoclonal antibody diluted 1:400 in 1% BSA/PBS. After washing three times in PBS for 5 min each time, the sections were treated with the Envision kit (DAKO, Tokyo, Japan) as follows: The sections were incubated for 16 h at 4 °C with a secondary goat-antimouse Immunoglobulin G (IgG; DAKO) conjugated to horseradish peroxidase (HRP, DAKO). After washing three times in PBS for 5 min each time, the sections were developed with 0.03% 3,3-diaminobenzidine tetrahydrochloride (DAKO) solution containing 0.005% H_2_O_2_. After visualization, sections were washed in water for 5 min and counterstained with hemotoxylin for 10 s. After washing in water for 10 min, the sections were dehydrated with ethanol and penetrated with xylene. The sections were examined with an AX80 microscope (Olympus Inc.). Peptide absorption tests of both monoclonal antibodies were performed.

### Real-time quantitative PCR

Retinal expression of *Rom1* and *Prph2* mRNA was measured by real-time quantitative PCR (qPCR). The eyes of 8-week-old mice were enucleated, and the retinas were dissected from the eyecups in ice-cold RNAlater (Product number No.7020; Applied Biosystems). Total RNA was extracted from the retinas with TRIzol (Invitrogen). Real-time qPCR was performed by using the SYBR® Reverse-Transcriptase (RT)–PCR kit (product code RR045A; TaKaRa, Shiga, Japan), according to the manufacturer’s protocol. Briefly, single-stranded cDNA was synthesized from each RNA sample with Rnase H free MMLV RTase (Takara) and random 6-mers on a PCR Thermal Cycler GP (TaKaRa). The cDNA obtained was amplified on a Smart Cycler II rapid DNA amplification system (Cepheid, TaKaRa) with a SYBR® RT–PCR kit, which contains SYBR Premix Ex Taq, SYBR Green I, and deoxyribonucleotide triphosphates (dNTPs). The PCR reaction was run under the following conditions: denaturation at 95 °C for 10 s, followed by 45 cycles of 95 °C for 5 s, and 60 °C for 20 s. The levels of glyceraldehyde-3-phosphate dehydrogenase (*Gapdh*) mRNA and rhodopsin (*Rho*) mRNA were used as endogenous controls for each sample. The mRNA levels in each sample were determined by using a calibration curve. Separate calibration (standard) curves for each gene were constructed by using serial dilutions of mRNA from a mouse retina of the same strain that had not been exposed to ENU. Primer sets for amplifying each gene were chosen from exons separated by an intron. The primer sets used in this analysis were: for *Rom1*, forward primer 5′-GCC ATG AAG TGC TGC TGG AA-3′, reverse primer 5′-GTC TGC AAA TAC CGC AAA CCA AG-3′; for *Prph2*, forward primer 5′-ACC ATC GAC ATG CTC CAG ATT G-3′, reverse primer 5′-CCC ATC CAC GTT GCT CTT GA-3′; for *Gapdh*, forward primer 5′-AAA TGG TGA AGG TCG GTG TG-3′, reverse primer 5′-TGA AGG GGT CGT TGA TGG-3′; and for *Rho*, forward primer 5′-TTG GGC CCA CAG GCT GTA A-3′, reverse primer 5′-CCG AAG CGG AAG TTG CTC A-3′.

### Western blotting

Retinas were dissected from eyes collected from 3-week-old mice and homogenized by sonication in ice-cold buffer (20 mM Tris [pH. 7.6], 137 mM NaCl, 10% glycerol, 1% NP-40) containing protease inhibitor (1 mM phenylmethylsulfonyl fluoride; Sigma-Aldrich, Tokyo, Japan, 10 μg/ml aprotinin ; Sigma-Aldrich, Tokyo, Japan, 1 μg/ml leupeptin ; Peptide Institute, Inc., Osaka, Japan). After centrifugation at 10,000× g for 15 min at 4 °C, the supernatant was collected and protein concentrations were determined with a bicinchoninic acid (BCA) protein assay kit (Product number 23225; Thermo Fisher Scientific, Rockford, IL), with BSA used as a standard. For western blotting of Rom1 and Prph2, 10 µg of the retinal protein was mixed with the same volume of 2× loading buffer (140 mM Tris [pH. 6.8], 140 mM NaCl, 60% glycerol, 4% sodium dodecyl sulfate, 0.02% bromophenol blue, 5.0% β-mercaptoethanol) and boiled for 10 min. The prepared protein samples were electrophoresed by 12.5% sodium dodecyl sulfate–PAGE and transferred to Immobilon transfer membranes (polyvinylidene difluoride membrane, Millipore, Tokyo, Japan). The membranes were blocked with 3% BSA in Tris-Buffered Saline Tween-20 (TBST; 20 mM Tris [pH. 7.6], 137 mM NaCl, 0.1% Tween-20) for 60 min and incubated for 60 min with mouse Rom1 antibody diluted 1:50 or mouse Prph2 antibody diluted 1:30 in TBST. After washing with TBST three times for 10 min each, the membranes were incubated with goat antimouse IgG conjugated to HRP (Product No. 62–6320; ZYMED Laboratories, San Francisco, CA). After washing with TBST three times for 10 min each, the membranes were immersed in Immobilon Western Chemiluminescent HRP Substrate (Millipore), and then immediately exposed to an LAS-3000 imaging system (FUJIFILM, Tokyo, Japan) to visualize the immunoreactive signals. Signal intensities were densitometrically quantitated with the ImageJ 1.41 software program (National Institutes of Health, Bethesda, MD). For western blotting of Rho, 0.1µg of the retinal protein was mixed with the same volume of 2× loading buffer. Samples were electrophoresed and transferred to a membrane without boiling. Mouse antibovine rhodopsin monoclonal antibody (Product No. MAB5356, clone name Rho 1D4; Chemicon, Temecula, CA) was diluted 1: 4,000 in high-salt TBST (20 mM Tris [pH.7.6], 1.14 M NaCl, 0.1% Tween-20), and immunoreaction with Rho antibody was allowed to proceed under the same conditions as with the Rom1 and Prph2 antibodies in high-salt TBST. The band between 25 kDa and 37 kDa was measured as the rhodopsin signal. The protein level in each sample was standardized to the level of β-actin, which was detected with a human β-actin monoclonal antibody conjugated to HRP (Product No. ab20272; Cosmo Bio Co., Ltd., Tokyo, Japan).

### Statistical analysis

The results were statistically analyzed by a one-way analysis of variance (ANOVA) and expressed as means±standard error when comparing groups. All multiple pairwise comparisons were analyzed by the Tukey–Kramer multiple comparisons test. A p value <0.05 was considered statistically significant.

## Results

### Discovery of a dominant mutant in *Rom1* generated by N-ethyl-N-nitrosourea mutagenesis

**Figure 1:**
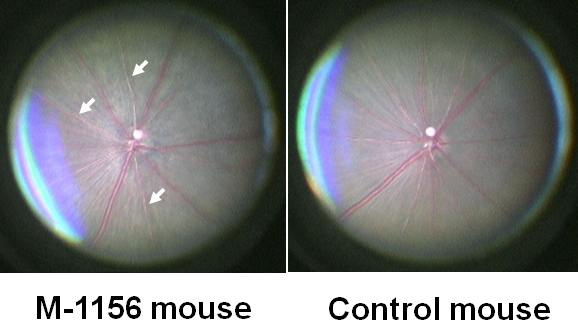
Appearance of the fundus of an *M-1156* mouse and a control mouse. At 45 weeks of age, the retinas of *M-1156* mice were mottled and their arteries (indicated by arrows) were narrower than in the retinas of control mice which were F1 mice from crossing DBA/2J mice and C57BL/6J mice; DBF1. *M-1156* mouse has same genetic background with DBF1.

During large-scale screening of ENU-mutagenized mice, we used a funduscopic camera to screen 2,515 first generation (G1) male mice for dominant abnormal eye phenotypes. Identified phenodeviants, i.e., animals that exhibited abnormal phenotypes, were subsequently backcrossed to DBA/2J or C3H/HeJ mice for inheritance testing. The inheritance tests confirmed that some independent phenotypes were clearly transmitted to the next generation. A catalog of the independent phenotypes will be described by us in near future. Comparison of the appearance of the fundi of heterozygous *M-1156* mice with the wild-type mice showed that they were abnormal, and a mottled retina and narrowed arteries were observed in the abnormal fundi (Figure 1). The mutation exhibited a dominant inheritance pattern. No other abnormalities were observed in the *M-1156* mice.

**Figure 2:**
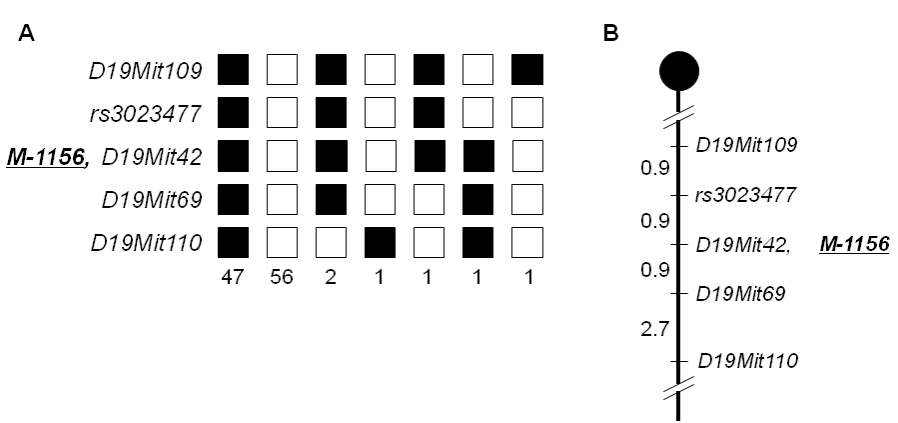
Haplotype analysis and genetic map of *M-1156*. **A**: Haplotype analysis of 109 backcross (N2) progeny from crossing *M-1156* and DBA/2J was shown. Genetic markers are listed on the left side (rs3023477 is a single nucleotide polymorphism [dbSNP]). Each column represents a chromosomal haplotype identified in the progeny. C57BL/6J alleles are indicated by black boxes, and DBA/2J alleles are indicated by white boxes. *M-1156* denotes the mutant locus. **B**: Genetic map around the causative gene was shown. The map distances (in cM) are shown on the left side of the map. The mutant locus *M-1156* was mapped close to D19Mit42 on mouse chromosome 19.

**Figure 3:**
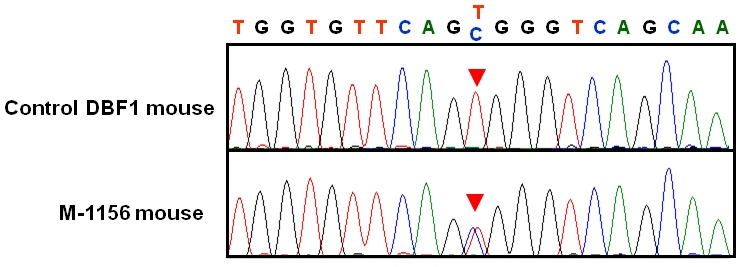
Sequence analysis of *Rom1*. The position of the point mutation is indicated by red arrowheads on sequence chromatograms from the Control DBF1 mouse (top) and the *M-1156* mouse (bottom). A T→C single base substitution at position 1,195 of *Rom1* (M96760, National Center for Biotechnology Information [NCBI]) was identified in the *M-1156* mouse line.

We mapped the mutant locus to the proximal region of chromosome 19 by linkage analysis of backcrossed progeny (Figure 2A). The mutated locus was mapped between rs3023477 and D19Mit69, close to D19Mit42 (Figure 2B). Although according to the public genome database (ensembl) there are more than 400 genes within the critical region, according to the PosMed web system *Rom1*, *Stx3*, and *Best1,* which encode rod outer segment membrane protein 1, syntaxin 3, and bestrophin 1, respectively, were strong candidate genes for retinal degeneration. We used genomic DNA from the mutant line to sequence their complete coding and flanking intron regions to determine whether the mutant phenotype was caused by a genetic alteration in *Rom1*, *Stx3*, or *Best1*. To exclude the possibility that base changes were pre-existing polymorphisms, we compared the sequences of the mutant mice with those of the wild-type (DBA/2J and C57BL/6J) mice. In addition, to exclude the possibility that base changes in *Prph2* contributed to the phenotype, we compared the sequences of the mutant mice with those of the wild-type (DBA/2J and C57BL/6J) mice. A single-base substitution, T→C, at position 1,195 of *Rom1* (M96760, National Center for Biotechnology Information [NCBI]) was identified in the *M-1156* mutant line (Figure 3), and the base change caused Trp to Arg amino acid substitution at position 182 of the translated protein. The base substitution was found in exon 1 of *Rom1*, and the amino acid substitution was located in the large intradiscal loop of Rom1. We designated the new *Rom1* mutant allele *Rom1^Rgsc1156^* (MGI accession number 3814074). No other alterations or polymorphisms in the *Rom1* coding region were found when mutant mice and wild-type mice were compared, nor were any sequence changes in *Stx3*, *Best1*, or *Prph2* detected in the mutant mice in a comparison with wild-type mice.

### Characterization of the phenotype of the *Rom1^Rgsc1156^* mutant

**Figure 4:**
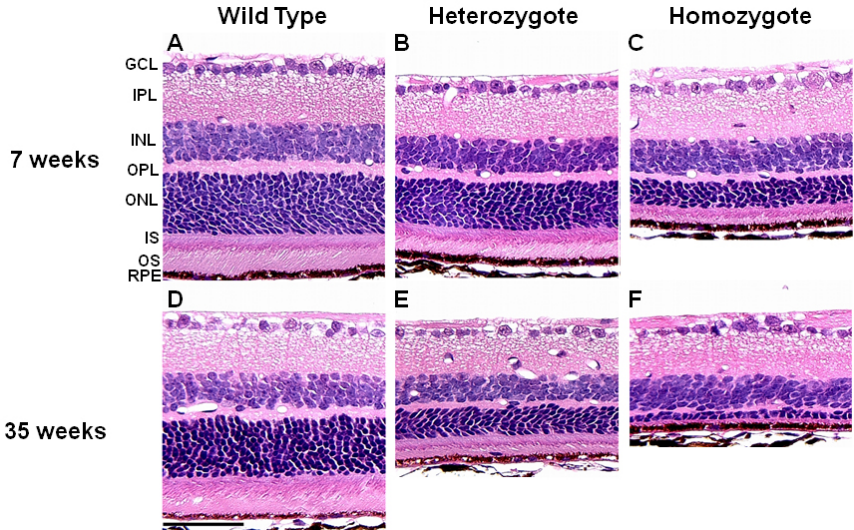
Photomicrographs of retinas from 7- (**A, B, C**) and 35- (**D, E, F**) week-old *Rom1^+/+^*, *Rom1^Rgsc1156/+^*, and *Rom1^Rgsc1156/Rgsc1156^* mice (n=3 or 4 each). The outer nuclear layer of the heterozygotes (**B, E**) was thinner and the photoreceptor outer segments were shorter than in wild-type mice (**A, D**). The alterations were even greater in the homozygotes (**C, F**). Differences were more marked at 35 weeks of age than at 7 weeks of age. GCL, IPL, INL, OPL, ONL, IS, OS, and RPE correspond to ganglion cell layer, inner plexiform layer, inner nuclear layer, outer plexiform layer, outer nuclear layer, inner segment, outer segment, and retinal pigment epithelium. The scale bar represents 50 µm.

**Figure 5:**
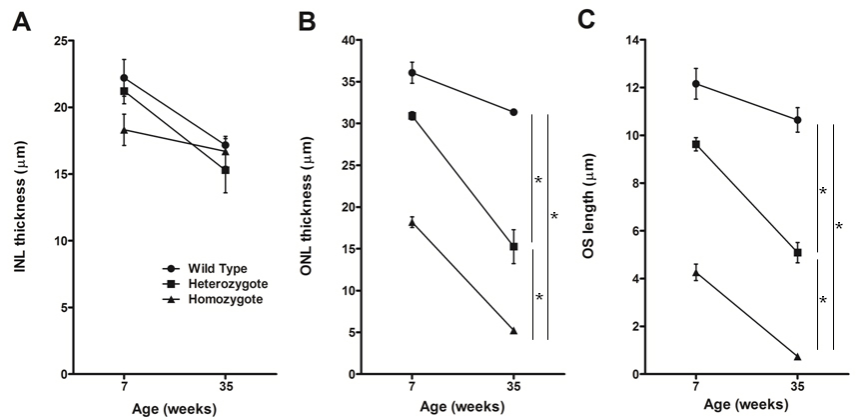
Inner nuclear layer (INL) and outer nuclear layer (ONL) thickness and outer segment (OS) length in the central retina. **A**: INL thickness did not significantly differ among the genotypes at the any age examined. ONL thickness (**B**) and OS length (**C**) significantly differed among the genotypes at every age examined (* indicates p<0.05, Tukey–Kramer multiple-comparisons test). At 35 weeks of age, OS length in the homozygotes was too short to be measured by our method. We examined a single eye of three or four animals of each genotype at each age. Data shown are means±standard error.

Careful examination of photomicrographs of the retinas of *Rom1^Rgsc1156^* heterozygotes and *Rom1^Rgsc1156^* homozygotes (n=3 or 4 each; Figure 4) revealed that the outer nuclear layer was thinner and the outer segments of the photoreceptors were shorter in the heterozygotes than in wild-type mice. The alterations were more marked in homozygotes than in heterozygotes, and they progressed from 7 weeks of age to 35 weeks of age. There were only one or two rows of nuclei in the outer nuclear layer of 35-week-old homozygotes, and the inner and outer segments were almost entirely absent. There were no significant differences among the genotypes in the thickness of the inner nuclear layer at any age examined, but there were significant differences in the thickness of the outer nuclear layer and the length of the outer segments of the photoreceptors (Figure 5).

**Figure 6:**
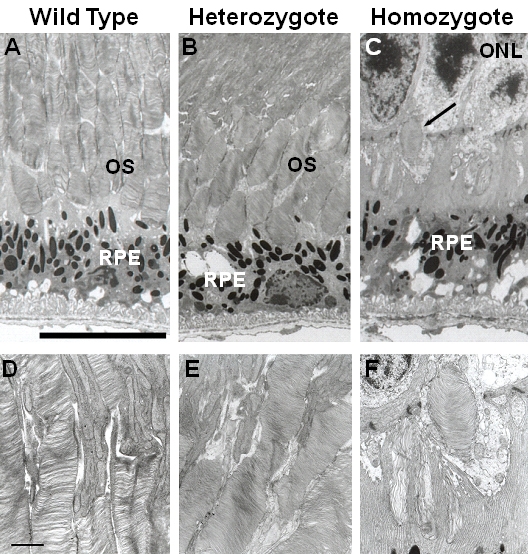
Electron micrographs of retinas from 35-week-old *Rom1^+/+^*, *Rom1^Rgsc1156/+^*, and *Rom1^Rgsc1156/Rgsc1156^* mice (n=3 each). The bar represents 10 µm in **A, B,** and **C** and 1 µm in **D, E,** and **F**. The photoreceptor outer segments were shorter in the heterozygotes (**B**) than in the wild-type mice (**A**). Remnants of the photoreceptor outer segments were observed in 35-week-old homozygotes (**C**, arrow). At high magnification the diameters of the discs clearly varied in the heterozygotes (**E**), and the discs of the heterozygotes were more compactly stacked than in the wild-type (**D**). (**D-F**) are high-magnified views of outer segment in (**A-C**), respectively.

Transmission electron microscopy was used to examine the ultrastructure of photoreceptor outer segments (n=3 each; Figure 6). The outer segments of the photoreceptors were shorter in the heterozygotes than in the wild-type mice. Only remnants of the outer segments of the photoreceptors were observed in the homozygotes at 35 weeks of age. High magnification revealed that the outer segments of the photoreceptors of the heterozygotes were clearly disorganized (the disc diameters varied and the discs were more compactly arranged than in wild-type mice).

**Figure 7:**
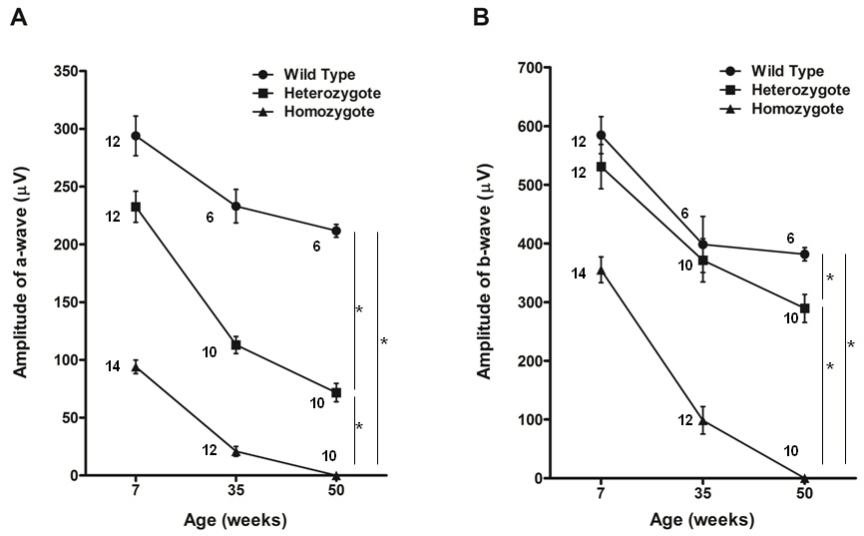
Single-flash electroretinograms of *Rom1^+/+^*, *Rom1^Rgsc1156/+^*, and *Rom1^Rgsc1156/Rgsc1156^* mice. **A**: The amplitude of the a-wave differed significantly among the genotypes at every age examined (* indicates p<0.05, Tukey–Kramer multiple-comparisons test). The reduction was most severe in the homozygotes. **B**: The amplitude of the b-wave also differed significantly among the genotypes (* indicates p<0.05, Tukey–Kramer multiple-comparisons test), although there were no significant differences between the wild type and heterozygotes at 7 and 35 weeks. The number of eyes analyzed in each group is indicated next to the symbols. Data shown are means±standard error.

**Figure 8:**
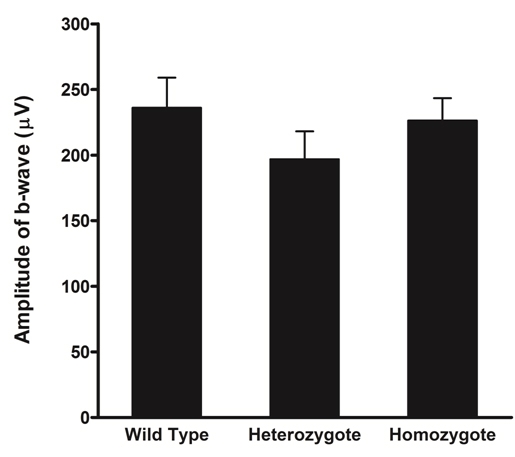
Photopic electroretinograms of 7-week-old *Rom1^+/+^*, *Rom1^Rgsc1156/+^*, and *Rom1^Rgsc1156/Rgsc1156^* mice (n=4 each). There were no significant differences in amplitude of the b-wave among the genotypes. Data shown are means±standard error.

Finally, we performed electroretinography to evaluate retinal function. Single-flash ERGs showed that the amplitudes of the a-wave in the mutant mice were significantly lower than in wild-type mice (Figure 7). The degree of reduction was more severe in the homozygotes than in the heterozygotes and was greater in 35-week-old mice than in 7-week-old mice. In contrast, there were no significant differences among the genotypes in the amplitude of the b-wave of the photopic electroretinograms (Figure 8).

### Expression of *Rom1* and *Prph2* in the *Rom1^Rgsc1156^* mutant

To examine whether the T→C single-base substitution in exon1 induces splice site changes, we sequenced retinal cDNA from both wild-type mice and *Rom1^Rgsc1156^* homozygotes. The mutant allele was expressed, and there were no splice site changes in *Rom1^Rgsc1156^* mutants (data not shown).

**Figure 9:**
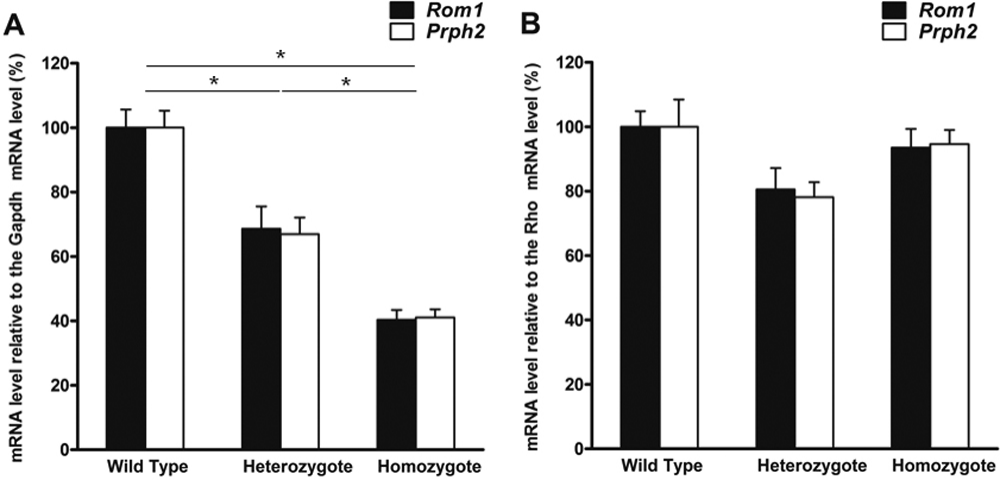
Expression levels of *Rom1* and *Prph2* in mouse retinas from 8-week-old *Rom1^+/+^, Rom1^Rgsc1156/+^*, and *Rom1^Rgsc1156/Rgsc1156^* mice (n=3 each). **A**: *Rom1* and *Prph2* mRNA levels are expressed relative to glyceraldehyde-3-phosphate dehydrogenase (*Gapdh*) mRNA level. The relative *Rom1* and *Prph2* mRNA levels differed significantly among the genotypes (* indicates p<0.05, Tukey–Kramer multiple-comparisons test). **B**: *Rom1* and *Prph2* mRNA levels are expressed relative to the *rhodopsin* (*Rho*) mRNA level. No significant differences in relative *Rom1* or *Prph2* mRNA levels were observed among the genotypes. Data shown are means±standard error. The data were normalized to wild-type values, which were set equal to 100%.

We also quantified the expression levels of both *Rom1* and *Prph2* in wild-type mice and *Rom1^Rgsc1156^* mutants by performing real-time PCR on samples from 8-week-old mice. Normalization of expression of *Rom1* to expression of *Gapdh* revealed that an expression level in *Rom1^Rgsc1156^* heterozygotes was 68.6±8.7% (mean±standard error) of the level in the wild type, whereas the expression level in homozygotes was 40.4±3.9% of the wild-type level (Figure 9A). Expression of *Prph2* relative to *Gapdh* in heterozygotes and homozygotes was 66.9±6.9% and 41.0±3.3%, respectively, of the level in the wild type. *Rom1* and *Prph2* mRNA levels relative to *Gapdh* mRNA differed significantly among the genotypes. When expression of *Rom1* was normalized to *Rho*, the expression level in *Rom1^Rgsc1156^* heterozygotes was 80.5±7.0% of the wild-type level, whereas in homozygotes the expression level was 93.5±6.2% of the wild-type level (Figure 9B). The relative expression of *Prph2* to *Rho* in heterozygotes and homozygotes was 78.1±5.0% and 94.6±4.6%, respectively, of expression in the wild type. No significant differences in *Rom1* or *Prph2* mRNA levels relative to *Rho* mRNA were detected among the genotypes.

### Production of Rom1 and Prph2 in the *Rom1^Rgsc1156^* mutant

**Figure 10:**
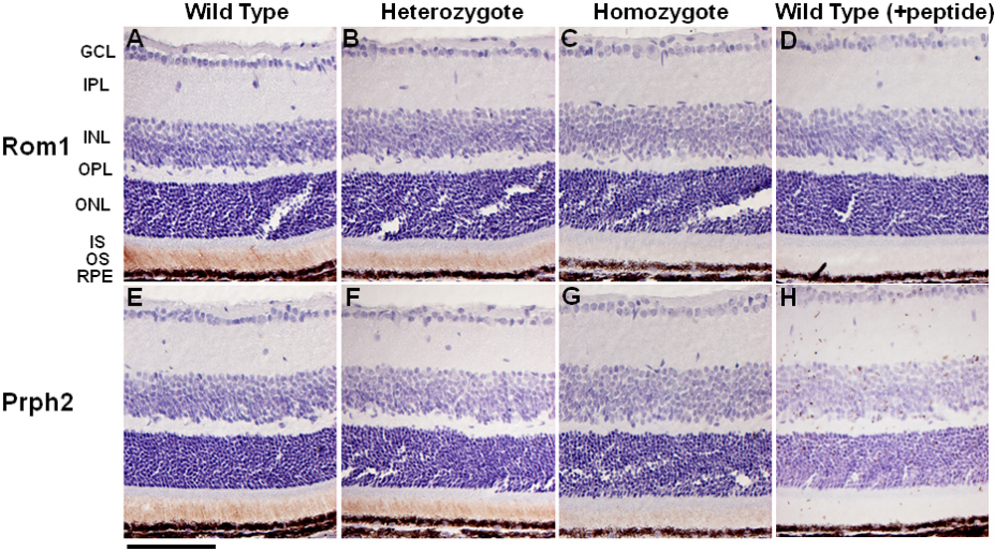
Immunohistochemical localization of retinal outer segment membrane protein 1 (Rom1) and peripherin/rds (Prph2) in the retinas of 3-week-old *Rom1^+/+^*, *Rom1^Rgsc1156/+^*, and *Rom1^Rgsc1156/Rgsc1156^* mice. Rom1 immunoreactivity was decreased in the outer segments of the heterozygotes (**B**) in comparison with wild-type mice (**A**), and Rom1 immunoreactivity was markedly reduced in the homozygotes (**C**). Prph2 immunoreactivity in the outer segment was also decreased (**E, F, G**). No signals were detected in the retinas of wild-type mice when peptide absorption tests were performed (**D, H**). The bar represents 50 µm. GCL, IPL, INL, OPL, ONL, IS, OS, and RPE refer to ganglion cell layer, inner plexiform layer, inner nuclear layer, outer plexiform layer, outer nuclear layer, inner segment, outer segment, and retinal pigment epithelium.

To investigate the production of Rom1 and Prph2 in *Rom1^Rgsc1156^* mutants, we performed immunohistochemistry and western blotting on samples obtained from mice at 3 weeks of age when there was still little change in the thickness of the outer nuclear layer and the length of the inner and outer segments of the photoreceptors at the light microscopic level. Rom1 immunoreactivity in the retina was decreased in heterozygotes compared to wild-type mice (Figure 10B), and Rom1 immunoreactivity was markedly reduced in the homozygotes (Figure 10C). Prph2 immunoreactivity was similarly reduced in both the heterozygotes and homozygotes (Figure 10F,G).

**Figure 11:**
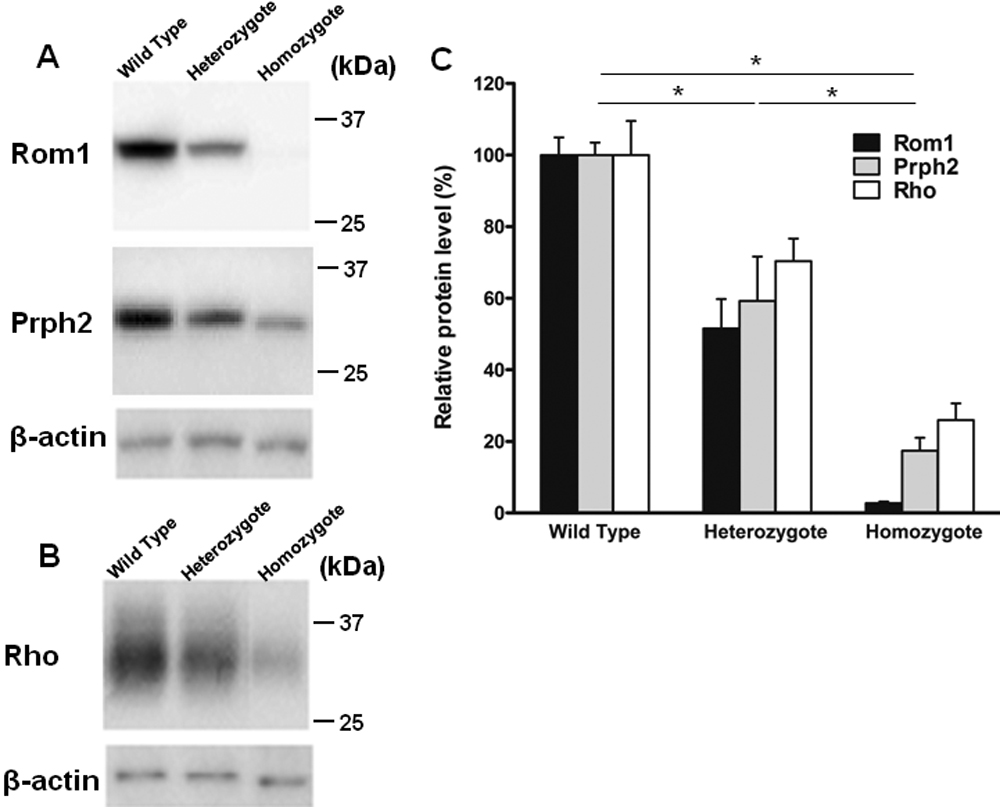
Western blot analysis of retinal outer segment membrane protein 1 (Rom1), Prph2, and Rho in retinas from 3-week-old *Rom1^+/+^, Rom1^Rgsc1156/+^,* and *Rom1^Rgsc1156/Rgsc1156^* mice (n=6 each). **A**: Western blots of 10 µg of retinal protein with antibodies to Rom1, Prph2, and β-actin. The stripped membrane was reprobed with antibodies to Prph2 and β-actin. **B**: Western blots of 0.1 µg of retinal protein with antibodies to Rho and β-actin. The stripped membrane was reprobed with β-actin antibodies. **C**: Semiquantitative analysis of Rom1, Prph2, and Rho levels. The western blot band intensities were measured with ImageJ software. Rom1, Prph2, and Rho protein levels were normalized to β-actin levels. The intensity (mean±standard error) was normalized to wild-type values, which were set equal to 100%. Protein levels differed significantly among the genotypes (* indicates p<0.05, Tukey–Kramer multiple-comparisons test).

Western blot analysis showed a decreased density of Rom1 and Prph2 from the *Rom1^Rgsc1156^* mutants (Figure 11A), and the density of Rho from the *Rom1^Rgsc1156^* mutants was also decreased (Figure 11B). Semiquantitative analysis by densitometry showed that Rom1 immunoreactivity in *Rom1^Rgsc1156^* heterozygotes and homozygotes was 51.6±8.2% and 2.8±0.3%, respectively, of that in wild-type mice (Figure 11C). The immunoreactivity of Prph2 from *Rom1^Rgsc1156^* heterozygotes and homozygotes was 59.3±12.4% and 17.4±3.6%, respectively, of the immunoreactivity of Prph2 from wild-type mice. The immunoreactivity of Rho from the *Rom1^Rgsc1156^* heterozygotes and homozygotes was 70.4±6.3% and 25.9±4.7%, respectively, of the immunoreactivity of the Rho from wild-type mice. The level of each protein differed significantly among the genotypes.

## Discussion

### Retinal degeneration in *Rom1^Rgsc1156^* mutants

*Rom1^Rgsc1156^* mutants have a T→C single base substitution at position 1,195 of *Rom1* (M96760, NCBI) and no mutations in *Prph2.* Sequence analysis of retinal cDNA around the mutation showed that the mutant allele was expressed and that the mutation did not alter the splice site. The results of the real-time PCR suggested that the decrease in *Rom1* and *Prph2* expression relative to *Gapdh* expression was attributable to the decrease in thickness of the outer nuclear layer because there were no significant differences in *Rom1* or *Prph2* mRNA levels among the genotypes after normalization to *Rho* mRNA. In short, since the total expression levels of *Rom1*, *Prph2,* and *Rho* in the mutant appear to be associated with the thickness of the outer nuclear layer, a protein with an Arg substituted for Trp at position 182 of the normal Rom1 protein should be produced.

Immunohistochemistry at 3 weeks of age showed that the immunoreactivity of Prph2 as well as Rom1 was reduced in *Rom1^Rgsc1156^* mutants, and western blot analysis demonstrated that the Rom1 and Prph2 levels in *Rom1^Rgsc1156^* mutants were significantly decreased. Interestingly, the Rho level was also decreased in *Rom1^Rgsc1156^* mutants at 3 weeks of age. A study of knockout and transgenic mice showed that retinal degeneration occurs when the combined level of both Rom1 and Prph2 is only about 60% of their combined level in wild-type mice [32]. Our semiquantitative analysis of western blots demonstrated that the combined level of both Rom1 and Prph2 was already below that percentage in *Rom1^Rgsc1156^* mutants at 3 weeks of age, suggesting that photoreceptor degeneration in *Rom1^Rgsc1156^* mutants occurred at least by 3 weeks of age. Why was Prph2 decreased in *Rom1^Rgsc1156^* mutants? Prph2 and Rom1 interact noncovalently via their large intradiscal loops to produce homotetrameric and heterotetrameric core complexes [33-35]. Since the Trp^182^ of Rom1 is localized in the intradiscal loop, it may induce unstable heterotetramers and homotetramers in *Rom1^Rgsc1156^* mutants. There is little Rom1 or Prph2 immunoreactivity in the inner segment of *Rom1^Rgsc1156^* mutants, and immunoreactivity for both proteins is decreased in the outer segment in a genotype-dependent manner, suggesting that mutant Rom1–Prph2 heterotetramers and mutant Rom1 homotetramers are degraded immediately after subunit assembly. Unfortunately, because of the small amount of mutant Rom1 in the homozygotes, our immunoprecipitation analysis failed to show that mutant Rom1 binds Prph2. In vitro biochemical analyses of subunit assembly may elucidate which core complexes are components in *Rom1^Rgsc1156^* mutants.

The ERGs showed significant differences among the genotypes in the amplitude of the a-wave in single-flash ERGs but not in the amplitude of the b-wave in photopic ERGs. These findings suggested that there was little effect on the function of cone photoreceptors in *Rom1^Rgsc1156^* mutants at 7 weeks of age but that there was significant deterioration of the function of rod photoreceptors in *Rom1^Rgsc1156^* mutants. Rod photoreceptor degeneration should mainly have occurred in *Rom1^Rgsc1156^* mutants because Rom1 is produced exclusively in rod photoreceptors.

All of the above findings taken together suggest that Trp182Arg mutant Rom1 decreases the levels of wild-type Prph2 and Rom1, which in turn results in a reduced level of Prph2-containing tetramers in the disc rim region and ultimately results in unstable disorganized outer segments and photoreceptor degeneration [34,36-38].

### Phenotypic severity in *Rom1^Rgsc1156^* mutants

Light microscopy showed that the thickness of the outer nuclear layer in 7-week-old *Rom1^Rgsc1156/+^* mice was almost the same as in 2-month-old *rds*/+ mice [39]; however, the electron microscopic appearance of the outer segments was extremely different. The disc diameters of the *Rom1^Rgsc1156/+^* mice varied and their discs were compactly stacked, while the outer segments of the *rds*/+ mice were whorls [7,40]. The length of the outer segments in the *rds*/+ mice was much shorter than in the in *Rom1^Rgsc1156/+^* mice and their length in *Rom1*^+/−^ mice was almost the same as their length in wild-type mice. According to the previous results [32], the levels of Prph2 and Rom1 in *Rom1*^+/−^ mice were 106±4% and 58±2%, respectively, of their level in the wild type, whereas their levels in *rds*/+ mice were 26±2% and 67±2%, respectively. In *Rom1^Rgsc1156/+^* mice, the levels of Prph2 and Rom1 were 59.3±12.4% and 51.6±8.2%, respectively. Taken together, these results suggest that the phenotypic severity in outer segments is due to the amount of Prph2-containing tetramers, which are required for higher order disulfide-linked oligomer formation because the level of oligomers is important for stable photoreceptor disc formation [36,37].

### *Rom1^Rgsc1156^* mutants can serve as a new animal model of human retinitis pigmentosa

Sequence alterations in human *ROM1* associated with RP have been reported. The three *ROM1* mutations (p.Gly80fs, p.Leu114fs, p.Gly113Glu) are manifested by digenic RP in combination with a Leu185Pro mutation in *PRPH2*, and only one of these mutations has an essentially normal phenotype [15,16]. The p.Gly80fs and p.Leu114fs mutations result in frameshifts early in the coding region and a downstream premature stop at codon 131, suggesting that these mutations do not encode a functional protein and therefore are null alleles. Production of the p.Gly113Glu Rom1 protein is 20-fold lower than production of wild-type Rom1 when expressed in COS-1 cells, indicating that a mutation resulting in p.Gly113Glu behaves as a null allele [37]. Biochemical studies have demonstrated that the p.Leu185Pro Prph2 mutant assembles with wild-type Prph2 and Rom1 to form core tetramers, but unlike wild-type Prph2 is unable to self-assemble into the core homotetramers that are required for higher order disulfide-linked oligomer formation [34,37]. Based on these findings, it seems likely that the pathogenesis of digenic RP is attributable to a large decrease in the amount of core complexes, such as Prph2 homotetramers and Prph2-Rom1 heterotetramers.

Two amino acid substitutions (p.Pro60Thr and p.Thr108Met) in a single allele of *ROM1* in an autosomal dominant RP patient and three different single amino acid substitutions (p.Gly75Asp, p.Leu114fs, p.Arg242Glu) in patients with sporadic RP have been reported [41-43]. None of these substitutions has been concluded to be pathogenic because the pedigrees reported have been so small that statistically significant co-segregation analyses are limited. Loss of one allele of *Rom1* in mice does not induce photoreceptor degeneration, implying that p.Leu114fs is unlikely to be pathogenic in humans [5].

In conclusion, the evidence is convincing that the Trp182Arg substitution in *Rom1^Rgsc1156^* mutants causes retinal degeneration, suggesting that mutations in the large intradiscal loop of Rom1 may affect the formation of homotetrameric and heterotetrameric core complexes and their stability. The *Rom1^Rgsc1156^* mutant will not only provide additional information about subunit assembly but will also serve as a new animal model of human autosomal dominant RP. The prevalence of nondigenic dominant *ROM1* mutations is less than 1% [43,44], but Rom1 may play a more important role in stabilizing the core complex than previously expected.
